# Inexpensive Bismuth-Film Electrode Supported on Pencil-Lead Graphite for Determination of Pb(II) and Cd(II) Ions by Anodic Stripping Voltammetry

**DOI:** 10.1155/2018/1473706

**Published:** 2018-10-08

**Authors:** Karen C. Bedin, Edson Y. Mitsuyasu, Amanda Ronix, André L. Cazetta, Osvaldo Pezoti, Vitor C. Almeida

**Affiliations:** Department of Chemistry, State University of Maringá, Av. Colombo 5790, CEP 87020-900 Maringá, PR, Brazil

## Abstract

The present work reports the development and application of bismuth-film electrode (BiFE), obtained by in situ method on the pencil-lead graphite surface, for simultaneous Cd(II) and Pb(II) determination at trace levels, as alternative to replace the mercury-film electrodes. Experimental factors, deposition time (*t*_*d*_), deposition potential (*E*_*d*_), and Bi(III) concentration (*C*_*Bi*_), were investigated by applying a 2^3^ factorial design using 0.10 mol/L acetate buffer solution (pH 4.5) as supporting electrolyte. The analysis conditions of the differential pulse technique were* t*_*d*_ = 250 s,* E*_*d*_ = -1.40 V, and* C*_*Bi*_ = 250 mg L^−1^. The validation of the method employing BiFE was accomplished by determination of merit figures. The detection limits were of 11.0 *μ*g L^−1^ for Cd(II) and 11.5 *μ*g L^−1^ for Pb(II), confirming that proposed method is attractive and suitable for heavy metals determination. Additionally, the BiFE developed was successfully applied for the Cd(II) and Pb(II) determination in wastewater sample of battery industry.

## 1. Introduction

Trace elements, such as Cu, Mo, Mn, and Zn, are considered essential for human health, while others as Hg, Pb, and Cd may accumulate in body tissues causing problems due to their toxicity [1, 2]. This accumulation is due to anthropogenic activities, associated with disposal of solid and liquid waste without proper treatment [3]. Considering the increasing industrial use of these heavy metals and their serious environmental and toxicological impacts, it is necessary to develop new analytical methods to determine them at trace levels [4, 5].

The analytical methods used for heavy metals determination include spectrometry and electroanalytical techniques. Atomic absorption spectrometry (AAS) is the most applied technique for metals determination, since it provides satisfactory sensitivity, high selectivity, and relatively low cost equipment. However, this technique has the disadvantage of not allowing simultaneous determination of chemical elements [6]. The inductively coupled plasma optical emission spectrometry (ICP-OES) and inductively coupled plasma mass spectrometry (ICP-MS) are techniques which allows multielement analysis; however, the high cost of installation and maintenance has restricted their use in research and routine analysis [7–10].

Anodic stripping voltammetry (ASV) is a versatile electroanalytical technique for the trace metals determination in various environmental, clinical and industrial samples [6]. Stripping methods are important in trace analysis because the electrodeposition step concentrates the analyte on the electrode surface, enabling its determination even in extremely low quantities with reasonable accuracy [11, 12].

During the past five decades, mercury-based electrodes were often applied in ASV for presenting an excellent analytical performance [13, 14]. However, due to the high toxicity of mercury and its compounds, the use of this metal in electrodes was restricted. Consequently, an intense research for less toxic and environmentally friendly materials than mercury has been promoted [15–17].

Bismuth-film electrodes were introduced in the last decade to replace the mercury-based electrodes used for the determination of trace heavy metals and organic compounds, due to very similar electrochemical properties of Bi and Hg, including alloying heavy metals with bismuth as an analogy to amalgamation at mercury [10, 18]. The bismuth-film electrode consists of a bismuth film deposited on a substrate, which can be Au [19], Pt [20], carbon paste [21], glassy carbon [22], carbon fiber [23], pyrolytic graphite [24], and oxides [3, 25, 26]. Moreover, different combinations also are possible as Bi_2_Te_3_-graphene oxide hybrid film [27] and bismuth-dispersed xerogel-based composite film [28], besides new methods for Bi_2_O_3_-electrode modification as spark discharge [29], proving that there are still issues to be explored in the development of bismuth-based electrodes. Performance of bismuth-film electrodes is similar to traditional mercury electrodes, in addition to the advantage of having negligible toxicity compared to them [30]. The bismuth-film electrode has been applied in voltammetric studies from the ex situ or in situ method, at potential range of -1.2 to 0 V. Positives potentials are not applied, since bismuth is completely removed from the substrate surface by oxidation under these conditions [14].

Carbon electrodes are used extensively as voltammetric sensors in various applications [31]. Mechanical pencils are inexpensive and alternative material to produce carbon electrodes, since they are commercially available with different diameters and hardness. This type of graphite has the advantage of not being fragile as the pyrolytic or paste carbon electrodes but is not hard as the glassy carbon electrode. In addition, graphite has good characteristics such as high electrical conductivity, quick and easy pretreatment, low cost, wide availability, minimum trace metals residue in its composition, and low background current [18].

Bond* et al. *[12] reported the use of a graphite electrode, which was optimized to anodic stripping analysis using mercury thin-film for Cd and Pb determination and the results were consistent with those obtained by analysis with glassy carbon electrode. The use of bismuth-film electrode supported on pencil graphite (BiFE) has been reported in the literature [3, 15, 32, 33]; however, investigation of manufacturing settings can provide better responses for analytical determination of metal ions.

The present work aimed to prepare a BiFE, investigating the experimental parameters by 2^3^ fractional factorial design, for the simultaneous determination of Pb(II) and Cd(II) ions by differential pulse anodic stripping voltammetry (DPASV). Additionally, the applicability of BiFE in DPASV was evaluated by the analytical figures of merit.

## 2. Materials and Methods

### 2.1. Reagents, Chemicals, and Samples

All chemicals used were of analytical grade and milli-Q water was used in the preparation of solutions. The Bi(III), Pb(II), and Cd(II) stock solutions (1000 mg L^−1^) were prepared from Bi(NO_3_)_2_.4H_2_O, Pb(NO_3_)_2_ and Cd(NO_3_)_2_.4H_2_O acquired from Sigma-Aldrich. The powder graphite, H_2_SO_4_, HNO_3_, K_4_Fe(CN)_6_, and CH_3_COOH were purchased from Merck. A 0.20 mol L^−1^ H_2_SO_4_ solution and a 0.10 mol L^−1^ acetate buffer (pH 4.50) were prepared as substrates electrolytes for the cyclic voltammetry studies of the prepared electrode and for Cd(II) and Pb(II) determination from DPASV, respectively. To evaluate the applicability of the electrode, wastewater samples of battery industry without any previous preparation were analyzed.

### 2.2. Apparatus

Voltammetric measurements were performed using a potentiostat (Autolab potentiostat/galvanostat GPES IME 663, PGSTAT302n 247V 50/60 Hz). The electrochemical cell coupled to potentiostat was composed of pencil-lead graphite working electrode (GE) or bismuth-film electrode supported on pencil-lead graphite (BiFE), Ag/AgCl as reference electrode and counter electrode of Pt wire.

### 2.3. Preparation of BiFE

**Figure 1:**
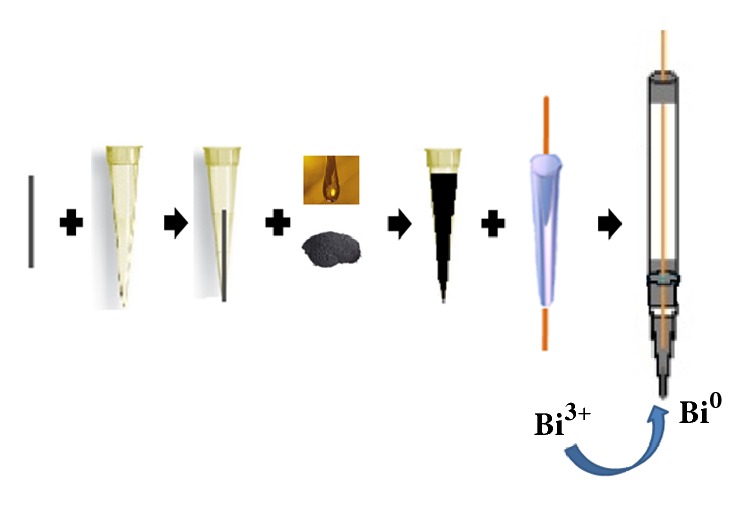
The assembly of pencil-lead graphite working electrode and bismuth-film formation.

Pencil-lead rods (Pentel Super, 2B, 0.7 mm in diameter) were used to prepare the GE and its assembly scheme is shown in Figure 1. Pencil-lead pieces of 2.5 cm were fitted to the micropipette tips, which were filled with carbon paste prepared by mixing graphite powder and mineral oil (Nujol) to promote the electric contact between the GE and copper wire. This wire was fixed in a glass tube that was connected to the micropipette tip, forming the electrode body. All connections were isolated using paste (cyanoacrylate), Teflon tape, and nonconductive epoxy resin. Due to this assembly, only the lower extremity of the pencil-lead rod (approximately 3.0 mm) is out of the micropipette tip and then available to act as the contact surface for film formation and determination of cations of interest. Since this surface was completely isolated with cyanoacrylate, a polishing step was necessary prior the experiments. Then, the polishing and the surface renewal of the electrodes were done by polishing them on a silk paper, until the base of the electrodes remained with metallic appearance.

The BiFE was prepared from the Bi(III) film formation on the GE surface by the cathode potential application (versus Ag/AgCl_sat_) by the in situ method. In this, the bismuth deposition occurred simultaneously with the electrochemical deposition of analytes. Moreover, the in situ procedure was chosen because it allows better adhesion of the Bi film to the GE surface, besides the obtaining of higher and better resolved peaks in the voltammograms, along with the higher sensitivity to Cd(II) and Pb(II) when compared to the ex situ method [34, 35].

### 2.4. Procedure

The cyclic voltammetry analysis was performed as a performance test of the GE prepared as working electrode, using 2.0 mol L^−1^ H_2_SO_4_ as the supporting electrolyte. Differential pulse anodic stripping voltammetry (DPASV) was carried out using standard Bi(III) solution (150 to 250 mg L^−1^) in the electrochemical cell to form the Bi film. The deposition time, deposition potential and Bi(III) concentration were investigated using acetate buffer solution (0.1 mol L^−1^) as supporting electrolyte and potential step of 1.95 mV, while the solution was stirred. Before each cycle a 30 s conditioning step at 300 mV (under stirring) was used to remove the bismuth excess and/or the target metals on the electrode surface. The DPASV measurements were performed from equilibrium time of 15 s, time modulation of 0.05 s, pulse interval time of 0.25 s, amplitude modulation of 25 mV, scan rate of 10 mV s^−1^, and being with different Cd(II) and Pb(II) concentrations.

### 2.5. Experimental Design

The 2^3^ factorial design was applied to investigate the factors: deposition time (*t*_*d*_), Bi(III) solution concentration (*C*_*Bi*_), and deposition potential (*E*_*d*_), represented in Table 1. The responses to the proposed design were the current peak areas of the ASV for Cd(II) and Pb(II) at concentrations of 48.3 to 233 *μ*g L^−1^. The selection of the experimental domain for each factor was determined by previous experiences and literature. Experimental design and data processing were performed using Design Expert 7.1.3 software.

## 3. Results and Discussion

### 3.1. Cyclic Voltammetry

**Figure 2:**
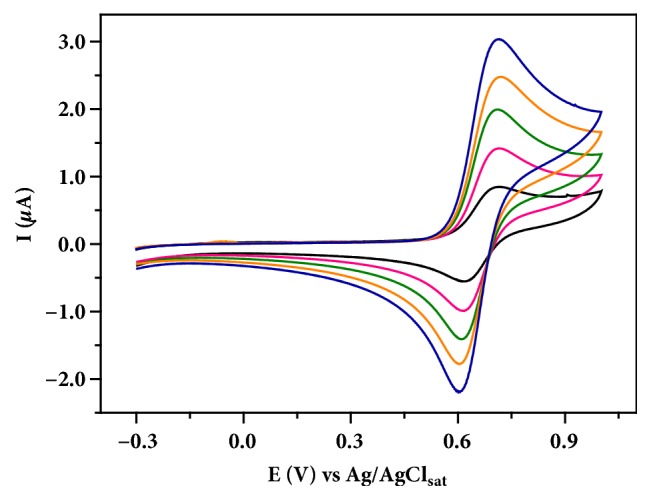
Cyclic voltammograms of potassium ferroferricyanide in 2.0 mol L^−1^ H_2_SO_4_ using GE (scan rate: 100 mV s^−1^).

The GE electrochemical performance was investigated from cyclic voltammetry measurements of the potassium ferroferricyanide (K_4_[Fe(CN)_6_] / K_3_[Fe(CN)_6_]) redox process.  Figure 2 shows the cyclic voltammograms of different K_4_[Fe(CN)_6_] concentrations. It can be seen that cathodic and anodic peaks occur in a short potential interval, indicating the reversibility of redox system.

Current values for anodic and cathodic peaks were proportional to the increase of the K_4_[Fe(CN)_6_] concentration. From the voltammograms of the cyclic voltammetry, it can be seen that the performance of the developed GE is comparable with the glassy carbon [12]. Additionally, a linear relationship between the peak current intensity and K_4_[Fe(CN)_6_] concentration was obtained and showed a linear regression equation of I_d_ = 3.16x10^−7^ + 4.18x10^−9^ C_K4[Fe(CN)6]_ and determination coefficient (R^2^) of 0.9992.

### 3.2. Anodic Stripping Voltammetry (ASV) with BiFE

**Figure 3:**
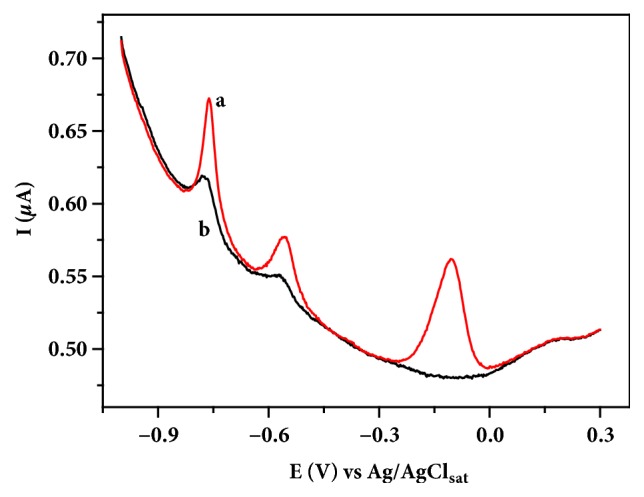
Differential pulse anodic stripping voltammograms obtained for Cd(II) and Pb(II) in 0.10 mol L^−1^ acetate buffer solution (pH 4.5) as supporting electrolyte; (a) 250 mg L^−1^ of Bi(III) dissolved in supporting electrolyte, (b) without Bi(III), determined with GE containing 142 *µ*g L^−1^ of Cd(II) and Pb(II) (*E*_*dp*_ = -1.40 V;* t*_*dp*_ = 250 s; potential step =1.95 mV; amplitude = 25 mV).

During the electrodeposition step in ASV method with BiFE, the codeposition of metallic ions present in the solution along with Bi^0^ on the graphite surface is possible [36]. Therefore, the film formation on the GE surface contributes to the improvement of the analytical signal corresponding to such ions, as shown in the Figure 3. It can be seen that the measurement performed with GE showed low current signals at -0.77 and -0.57 V, which are corresponding to Cd(II) and Pb(II), respectively. Applying BiFE, an increase in the analytic signal for the metal species present in solution can be observed, indicating a high sensitivity in the analysis. The highest current peak observed at -0.10 V corresponds to oxidation of bismuth film. According to the results, it can be inferred that other species with characteristic potential values higher than -0.10 V can be analyzed by the method.

### 3.3. Effect of the Factors Time (*t*_*d*_), Deposition Potential (*E*_*d*_), and Bi(III) Concentration (*C*_*Bi*_) on ASV Analyses

In order to investigate the effects of the factors* t*_*p*_*, E*_*d*_, and* C*_*Bi*_ on current measured for Cd(II) and Pb(II) determination using BiFE, a 2^3^ factorial design was carried out.  Table 2 shows the lower and superior levels values of the factors and its responses (current) obtained from the 8 ASV experiments.

**Figure 4:**
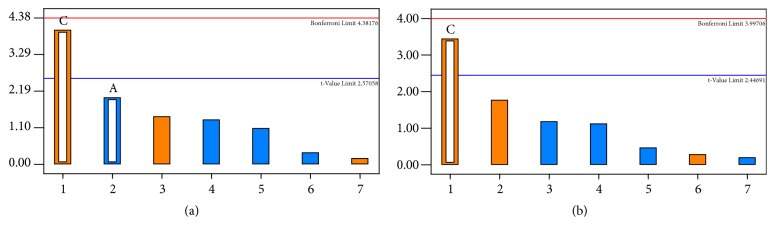
Pareto chart to evaluate the peaks area for Cd(II) (a) and Pb(II) (b), where* C*_*Bi*_ (A) and* t*_*d*_ (C).

Current measurements were made from the voltammograms obtained in potential values of -0.70 and -0.49 V for Cd(II) and Pb(II), respectively. As can be seen in Table 2, the lowest current values were observed for Cd(II), which ranged from 18.4 to 1.79 nA. The current peaks for Pb(II) determination ranged from 3.23 to 29.3 nA. Effects analyses were investigated by Pareto chart (Figure 4), showing the statistic t-test for each effect, where each bar represents the standard effect,* i.e.,* the estimated effect divided by its standard error and the t-critical value [37].

The factors represented by the bars that extend beyond the line are considered significant. According to the results, the* t*_*d*_ factor was significant for both metals causing a positive effect on the response. The other factors,* E*_*d*_ and* C*_*Bi*_, as well as the interaction effects between the factors, were not significant. This indicates that metal and film saturation on the electrode surface is not achieved at low time values. Therefore,* t*_*d*_ is the most important factor for the sensitivity of the technique using BiFE, which is in agreement with other works that reported the development of bismuth electrodes [18, 37, 38]. Thus, the optimized conditions selected for subsequent studies were those frequently reported in the literature: 250 s deposition time, Bi(III) concentration of 250 mg L^−1^, and -1.40 V deposition potential [3, 26, 38–40].

### 3.4. Analytical Method Development

**Figure 5:**
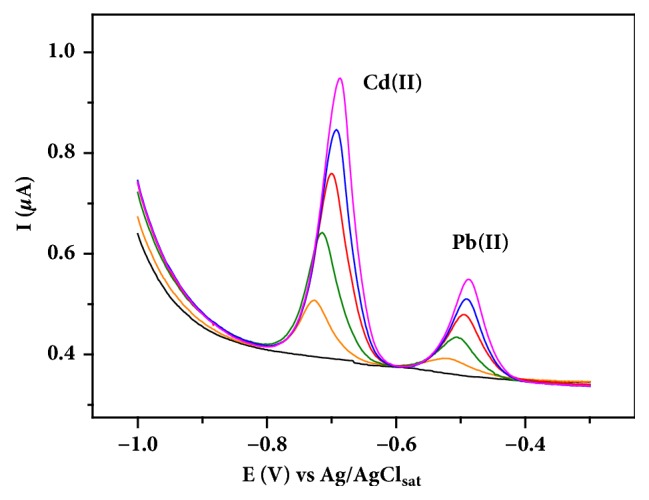
Differential pulse anodic stripping voltammograms for increasing concentrations (ranging from 48.3 to 233 *µ*g L^−1^) of Cd(II) and Pb(II) in 0.10 mol L^−1^ acetate buffer solution (pH 4.5) as supporting electrolyte using BiFE.

Analytical figures of merit were determined using the selected conditions (*t*_*d*_*, E*_*d*_, and* C*_*Bi*_) to evaluate the applicability of BiFE (film obtained by the in situ method) on the Cd(II) and Pb(II) determination.  Figure 5 shows differential pulse anodic stripping voltammograms for simultaneous determination of these ions using the BiFE. According to the figure, the two peaks corresponding to metal ions are well resolved and increase with increasing concentrations.

From the calibration curve of each chemical species, it can be demonstrated linearly between the current values and concentrations within the range of 48.3 to 233 *μ*g L^−1^ by the linear regression equation and determination coefficient of experimental data:* I*_*d*_ = 2.44x10^−3^ C_Cd_ + 4.29x10^−3^ and R^2^ = 0.9964 for Cd(II) and* I*_*d*_ = 8.35x10^−4^ C_Pb_ – 5.02x10^−3^ and R^2^ = 0.9954 for Pb(II).

#### 3.4.1. Detection and Quantification Limits

To evaluate the limit of detection (LOD) and limit of quantification (LOQ), five different blank solutions were used, which were analyzed in triplicate and the standard deviation (*S*_*B*_) of the means was calculated. The measurements were performed in 5.0 mL of blank solutions with 5.0 mL of acetate buffer solution, under the optimized conditions. The LOD and LOQ values were determined from the 3*S*_*b*_/*m* and 10*S*_*b*_/*m* relations, respectively, where* m* is the slope of the calibration curve of each chemical species. For Cd(II), the calculated values of LOD and LOQ were of 11.0 *μ*g L^−1^ and 36.8 *μ*g L^−1^, respectively. For Pb(II), the LOD was 11.5 *μ*g L^−1^ and LOQ was 38.2 *μ*g L^−1^. The LOD values obtained at levels of *μ*g L^−1^ are comparable with other studies involving modified electrodes. Serrano* et al.* [41] prepared modified graphite-epoxy composite electrodes which showed LOD of 2.40 and 4.70 *μ*g L^−1^ for Cd(II) and of 1.50 and 3.30 *μ*g L^−1^ for Pb(II), simultaneously determined. Kadara and Tothill [42] found LOD of 8.00 and 16.0 *μ*g L^−1^ for Pb(II) and Cd(II), respectively, using a screen-printed Bi_2_O_3_-modified electrode. Lezi* et al.* [43] employed screen-printed electrodes modified with five bismuth precursor compounds in Pb(II) and Cd(II) determination, obtaining LOD values of 0.90-1.40 and 1.10-3.20 *μ*g L^−1^, respectively. Therefore, low LOD values could be achieved by increasing the deposition time during the Bi film formation in the proposed method, once the deposition time showed a positive effect on the response by increasing the current intensity, as demonstrated in Table 2. Some studies in the literature corroborate this fact, as an example: Zhang* et al.* [35] employed a deposition time of 300 s during the in situ Bi deposition on working electrode and LOD of 0.02 *μ*g L^−1^ for Pb(II) and 0.01 *μ*g L^−1^ for Cd(II) were obtained; Demetriades* et al.* [18] prepared a bismuth-film electrode supported on pencil graphite with Bi deposition time of 600 s and LOD of 0.30 *μ*g L^−1^ for Cd(II) and 0.40 *μ*g L^−1^ for Pb(II) were determined.

#### 3.4.2. Repeatability

The repeatability (intraday) was evaluated from five measurements (in triplicate) of solutions containing 150 *μ*g L^−1^ Cd(II) or 150 *μ*g L^−1^ Pb(II) with 10.0 mL of acetate buffer solution, with just one BiFE. The mean of triplicates and the corresponding relative standard deviation (RSD) were calculated to obtain the analytical parameter. The repeatability values obtained, expressed as % RSD, were of 9.07% for Cd(II) and 11.6% for Pb(II), which are acceptable with respect to trace level determination of heavy metals.

#### 3.4.3. Reproducibility

The reproducibility was determined from measurements (in triplicate) of five different BiFEs for solutions of 150 *μ*g L^−1^ Cd(II) or 150 *μ*g L^−1^ Pb(II) with 10.0 mL of acetate buffer solution. This analytical parameter was calculated from RSD obtained from the mean value of measurements. The reproducibility values (expressed as the % RSD) achieved in this work were of 9.72% for Cd(II) and 7.87% for Pb(II) determination.

#### 3.4.4. Standard Addition Method

**Figure 6:**
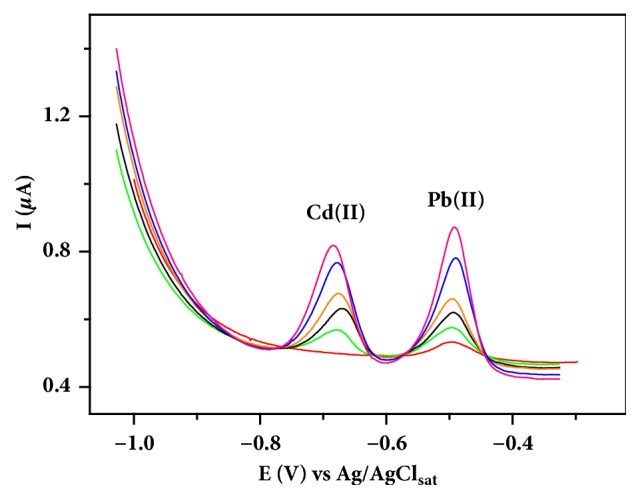
Differential pulse anodic stripping voltammograms for 1.00 mL wastewater sample in 9.00 mL acetate buffer solution (pH 4.5) with increasing Cd(II) and Pb(II) concentrations (48.3 to 233 *µ*g L^−1^) using BiFE.

The BiFE was applied in the determination of Cd(II) and Pb(II) in a wastewater sample from a battery industry. The sample was analyzed without any previous preparation and ions determination was performed by DPASV under optimized conditions.  Figure 6 shows the voltammograms obtained by the standard addition method for Cd(II) and Pb(II) determination in the sample. The curve was constructed using concentrations of Cd(II) and Pb(II) ranging from 48.3 to 233 *μ*g L^−1^.

**Figure 7:**
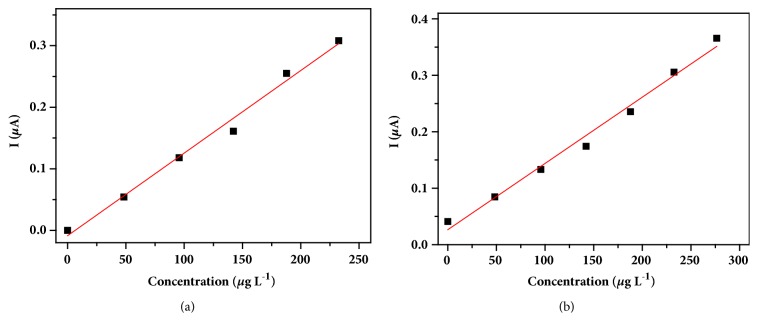
Calibration curves of standard addition method for Cd(II) (a) and Pb(II) (b).

The calibration curves for the standard addition method of each chemical species are shown in Figure 7. According to the figure, a good linearity between current signals and analytes concentrations can be observed. The linear regression equation and determination coefficient obtained were* I*_*d*_ = 1.36x10^−3^ C_Cd_ – 1.05x10^−2^ and R^2^ = 0.9873 for Cd(II) and* I*_*d*_ = 1.35x10^−3^ C_Pb_ + 1.79x10^−2^ and R^2^ = 0.9706 for Pb(II). The Cd(II) was not detected in the sample, while the Pb(II) determined concentration was of 48.8 *μ*g L^−1^. To compare these results with a reference method the wastewater sample was analyzed by graphite furnace atomic absorption spectrometry (GFAAS); Cd(II) was not detected and the Pb(II) determined concentration was 50.1 *μ*g L^−1^, consistent with the measurements using BiFE.

#### 3.4.5. Recovery Test

The recovery method was used in the battery industry wastewater sample, which was fortified with 190 *μ*g L^−1^ Cd(II) and 235 *μ*g L^−1^ Pb(II) solutions.  Table 3 shows the results of the recovery study in wastewater sample using BiFE and optimized experimental conditions. From the table, it can be noticed that the recovery for both metals was close to 100%, ranging from 96.9 to 108% for the addition levels studied. This indicates adequate precision and accuracy of the method employing BiFE for determination of heavy metals at trace levels.

## 4. Conclusions

The BiFE developed by in situ method for Pb(II) and Cd(II) simultaneous determination at trace levels showed an efficient analytical performance. Among the parameters investigated for differential pulse anodic stripping voltammetry, the deposition time was the most important factor for the technique sensitivity. From the optimized conditions, the BiFE exhibited a linear response in the range between 48.3 and 233 *μ*g L^−1^ with a detection limit of 11.0 *μ*g L^−1^ for Cd(II) and 11.5 *μ*g L^−1^ for Pb(II). Repeatability and reproducibility showed acceptable values for trace level determination of heavy metals. A wastewater sample of battery industry was used in the recovery method to evaluate the analytical application of BiFE on real samples. The recovery for both metals was close to 100%, demonstrating the precision and accuracy of the electrode developed. In this way, the results imply that BiFE can be useful in trace level determination of Cd(II) and Pb(II) in heavily polluted samples (industrial waste waters, aqueous sources from mining areas, etc.), thanks to its analytical performance, low cost, wide availability, easy preparation, and less time consuming.

## Figures and Tables

**Table 1:** Factors and respective lower and superior levels values.

Factors	Lower level (-)	Superior level (+)
Bi(III) concentration (mg L^−1^)	150	250
Deposition potential (V)	-1.50	-1.10
Deposition time (s)	100	250

**Table 2:** Experiments of 2^3^ factorial design and response values.

*Order*	*C*_*Bi*_ *(mg L*^*-1*^)	*E*_*d*_ *(V)*	*t*_*d*_ *(s)*	*I*_*Cd*(*II*)_ *(nA)*	*I*_*Pb*(*II*)_ *(nA)*
1	150	-1.50	100	5.31	7.92
2	150	-1.50	250	16.8	29.3
3	250	-1.50	100	4.71	9.26
4	150	-1.10	250	18.4	29.3
5	250	-1.50	250	7.02	14.9
6	250	-1.10	250	6.97	14.9
7	150	-1.10	100	2.75	3.23
8	250	-1.10	100	1.79	6.37

**Table 3:** Recovery of Cd(II) and Pb(II) in battery industry wastewater sample fortified in two concentration levels: (I) 190 *μ*g L^−1^ and (II) 235 *μ*g L^−1^.

Cd(II) (*μ*g L^−1^)			Recovery (%)		Pb(II) (*μ*g L^−1^)			Recovery (%)	
Blank	(I)	(II)	(I)	(II)	Blank	(I)	(II)	(I)	(II)

ND	205 ± 7.4	228 ± 8.5	108 ± 3.9	96.9 ± 3.6	15.1	190 ± 10.4	239 ± 12.2	100 ± 5.5	102 ± 5.2

## Data Availability

The authors confirm that all data created during this research, which are required to prove the results, are included within the article. In addition, questions about the data used to support the findings of this study are available from the corresponding author (vcalmeida@uem.br) upon request.

## References

[1] Wonsawat W., Chuanuwatanakul S., Dungchai W., Punrat E., Motomizu S., Chailapakul O. (2012). Graphene-carbon paste electrode for cadmium and lead ion monitoring in a flow-based system. *Talanta*.

[2] Serrano N., Díaz-Cruz J. M., Ariño C., Esteban M. (2010). Stripping analysis of heavy metals in tap water using the bismuth film electrode. *Analytical and Bioanalytical Chemistry*.

[3] Pokpas K., Zbeda S., Jahed N., Mohamed N., Baker P. G., Iwuoha E. I. (2014). Electrochemically reduced graphene oxide pencil-graphite in situ plated bismuth-film electrode for the determination of trace metals by anodic stripping voltammetry. *International Journal of Electrochemical Science*.

[4] Ayenimo J. G., Adeloju S. B. (2016). Rapid amperometric detection of trace metals by inhibition of an ultrathin polypyrrole-based glucose biosensor. *Talanta*.

[5] Fu L., Li X., Yu J., Ye J. (2013). Facile and Simultaneous Stripping Determination of Zinc, Cadmium and Lead on Disposable Multiwalled Carbon Nanotubes Modified Screen-Printed Electrode. *Electroanalysis*.

[6] Ping J., Wu J., Ying Y., Wang M., Liu G., Zhang M. (2011). Evaluation of trace heavy metal levels in soil samples using an ionic liquid modified carbon paste electrode. *Journal of Agricultural and Food Chemistry*.

[7] Fan F., Dou J., Ding A., Zhang K., Wang Y. (2013). Determination of lead by square wave anodic stripping voltammetry using an electrochemical sensor. *Analytical Sciences*.

[8] Ndlovu T., Arotiba O. A., Sampath S., Krause R. W., Mamba B. B. (2012). Electroanalysis of copper as a heavy metal pollutant in water using cobalt oxide modified exfoliated graphite electrode. *Physics and Chemistry of the Earth*.

[9] Prakash S., Shahi V. K. (2011). Improved sensitive detection of Pb2+ and Cd2+ in water samples at electrodeposited silver nanonuts on a glassy carbon electrode. *Analytical Methods*.

[10] Xu H., Zeng L., Huang D., Xian Y., Jin L. (2008). A Nafion-coated bismuth film electrode for the determination of heavy metals in vegetable using differential pulse anodic stripping voltammetry: An alternative to mercury-based electrodes. *Food Chemistry*.

[11] Keawkim K., Chuanuwatanakul S., Chailapakul O., Motomizu S. (2013). Determination of lead and cadmium in rice samples by sequential injection/anodic stripping voltammetry using a bismuth film/crown ether/Nafion modified screen-printed carbon electrode. *Food Control*.

[12] Bond A. M., Mahon P. J., Schiewe J., Vicente-Beckett V. (1997). An inexpensive and renewable pencil electrode for use in field-based stripping voltammetry. *Analytical Chemistry Acta*.

[13] Injang U., Noyrod P., Siangproh W., Dungchai W., Motomizu S., Chailapakul O. (2010). Determination of trace heavy metals in herbs by sequential injection analysis-anodic stripping voltammetry using screen-printed carbon nanotubes electrodes. *Analytica Chimica Acta*.

[14] Dornellas R. M., Franchini R. A. A., Aucelio R. Q. (2013). Determination of the fungicide picoxystrobin using anodic stripping voltammetry on a metal film modified glassy carbon electrode. *Electrochimica Acta*.

[15] Foster C. W., de Souza A. P., Metters J. P., Bertotti M., Banks C. E. (2015). Metallic modified (bismuth, antimony, tin and combinations thereof) film carbon electrodes. *Analyst*.

[16] Bernardelli J. K. B., Lapolli F. R., Da Silva Cruz C. M. G., Floriano J. B. (2011). Determination of zinc and cadmium with characterized electrodes of carbon and polyurethane modified by a bismuth film. *Materials Research*.

[17] Rehacek V., Hotovy I., Vojs M., Mika F. (2008). Bismuth film electrodes for heavy metals determination. *Microsystem Technologies*.

[18] Demetriades D., Economou A., Voulgaropoulos A. (2004). A study of pencil-lead bismuth-film electrodes for the determination of trace metals by anodic stripping voltammetry. *Analytica Chimica Acta*.

[19] Salles M. O., De Souza A. P. R., Naozuka J., De Oliveira P. V., Bertotti M. (2009). Bismuth modified gold microelectrode for Pb(II) determination in wine using alkaline medium. *Electroanalysis*.

[20] Švancara I., Baldrianová L., Tesařová E. (2006). Recent advances in anodic stripping voltammetry with bismuth-modified carbon paste electrodes. *Electroanalysis*.

[21] Wang J., Mo J.-W., Li S., Porter J. (2001). Comparison of oxygen-rich and mediator-based glucose-oxidase carbon-paste electrodes. *Analytica Chimica Acta*.

[22] Liu L., Ma Z., Zhu X., Zeng R., Tie S., Nan J. (2016). Electrochemical behavior and simultaneous determination of catechol, resorcinol, and hydroquinone using thermally reduced carbon nano-fragment modified glassy carbon electrode. *Analytical Methods*.

[23] Hutton E. A., Hočevar S. B., Ogorevc B. (2005). Ex situ preparation of bismuth film microelectrode for use in electrochemical stripping microanalysis. *Analytica Chimica Acta*.

[24] Lu M., Rees N. V., Compton R. G. (2012). Determination of Sb(V) Using Differential Pulse Anodic Stripping Voltammetry at an Unmodified Edge Plane Pyrolytic Graphite Electrode. *Electroanalysis*.

[25] Hull E., Piech R., Kubiak W. W. (2008). Iridium oxide film electrodes for anodic stripping voltammetry. *Electroanalysis*.

[26] Hwang G. H., Han W. K., Park J. S., Kang S. G. (2008). Determination of trace metals by anodic stripping voltammetry using a bismuth-modified carbon nanotube electrode. *Talanta*.

[27] Tseliou F., Avgeropoulos A., Falaras P., Prodromidis M. I. (2017). Low dimensional Bi2Te3-graphene oxide hybrid film-modified electrodes for ultra-sensitive stripping voltammetric detection of Pb(II) and Cd(II). *Electrochimica Acta*.

[28] Dimovasilis P. A., Prodromidis M. I. (2013). Bismuth-dispersed xerogel-based composite films for trace Pb(II) and Cd(II) voltammetric determination. *Analytica Chimica Acta*.

[29] Riman D., Jirovsky D., Hrbac J., Prodromidis M. I. (2015). Green and facile electrode modification by spark discharge: Bismuth oxide-screen printed electrodes for the screening of ultra-trace Cd(II) and Pb(II). *Electrochemistry Communications*.

[30] Frena M., Campestrini I., De Braga O. C., Spinelli A. (2011). In situ bismuth-film electrode for square-wave anodic stripping voltammetric determination of tin in biodiesel. *Electrochimica Acta*.

[31] Tavares P. H. C. P., Barbeira P. J. S. (2008). Influence of pencil lead hardness on voltammetric response of graphite reinforcement carbon electrodes. *Journal of Applied Electrochemistry*.

[32] Pierini G. D., Pistonesi M. F., Di Nezio M. S., Centurión M. E. (2016). A pencil-lead bismuth film electrode and chemometric tools for simultaneous determination of heavy metals in propolis samples. *Microchemical Journal*.

[33] Asadpour-Zeynali K., Najafi-Marandi P. (2011). Bismuth modified disposable pencil-lead electrode for simultaneous determination of 2-nitrophenol and 4-nitrophenol by net analyte signal standard addition method. *Electroanalysis*.

[34] Arduini F., Calvo J. Q., Palleschi G., Moscone D., Amine A. (2010). Bismuth-modified electrodes for lead detection. *TrAC - Trends in Analytical Chemistry*.

[35] Zhang X., Zhang Y., Ding D. (2016). On-site determination of Pb2+ and Cd2+ in seawater by double stripping voltammetry with bismuth-modified working electrodes. *Microchemical Journal*.

[36] Li D., Jia J., Wang J. (2010). A study on the electroanalytical performance of a bismuth film-coated and nafion-coated glassy carbon electrode in alkaline solutions. *Microchimica Acta*.

[37] Pinto L., Lemos S. G. (2013). Multivariate optimization of the voltammetric determination of Cd, Cu, Pb and Zn at bismuth film. Application to analysis of biodiesel. *Microchemical Journal*.

[38] Chen C., Niu X., Chai Y. (2013). Determination of lead(II) using screen-printed bismuth-antimony film electrode. *Electroanalysis*.

[39] Kachoosangi R. T., Banks C. E., Ji X., Compton R. G. (2007). Electroanalytical determination of cadmium(II) and lead(II) using an in-situ bismuth film modified edge plane pyrolytic graphite electrode. *Analytical Sciences*.

[40] Armstrong K. C., Tatum C. E., Dansby-Sparks R. N., Chambers J. Q., Xue Z.-L. (2010). Individual and simultaneous determination of lead, cadmium, and zinc by anodic stripping voltammetry at a bismuth bulk electrode. *Talanta*.

[41] Serrano N., González-Calabuig A., Del Valle M. (2015). Crown ether-modified electrodes for the simultaneous stripping voltammetric determination of Cd(II), Pb(II) and Cu(II). *Talanta*.

[42] Kadara R. O., Tothill I. E. (2008). Development of disposable bulk-modified screen-printed electrode based on bismuth oxide for stripping chronopotentiometric analysis of lead (II) and cadmium (II) in soil and water samples. *Analytica Chimica Acta*.

[43] Lezi N., Economou A., Dimovasilis P. A., Trikalitis P. N., Prodromidis M. I. (2012). Disposable screen-printed sensors modified with bismuth precursor compounds for the rapid voltammetric screening of trace Pb(II) and Cd(II). *Analytica Chimica Acta*.

